# Correction to: Conflict and COVID-19 in Yemen: beyond the humanitarian crisis

**DOI:** 10.1186/s12992-021-00750-z

**Published:** 2021-09-03

**Authors:** Mohammed Alsabri, Ayman Alhadheri, Luai M. Alsakkaf, Jennifer Cole

**Affiliations:** 1 Pediatrics, 1 Brookdale University Hospital and Medical center 1Brookdale Plaza, Brooklyn, NY 11212 USA; 2 Emergency Department, Al Thawra Modern General Hospital (TMGH), Sana’a City, Yemen; 3 Emergency Medicine, McLaren Oakland Hospital, 50 N. Perry St, Pontiac, MI 48342 USA; 4 Department of Geography, The Royal Holloway University of London, Egham Hill, Egham, Surrey, TW20 0EX UK

**Correction to: Global Health 17, 83 (2021)**

**https://doi.org/10.1186/s12992-021-00732-1**

Following publication of the original article [1], it was brought to our attention that the article had published with the following errors:

In the Abstract, line 2:

“The Yemeni people have been are left to fend for themselves” *should be “*The Yemeni people have been left to fend for themselves”.

On page 2

“The COVID-19 has pandemic shown what a modern global crisis looks like” *should be “*The COVID-19 pandemic has shown what a modern global crisis looks like”.

Lastly, in author details:

“Geography, The Royal Holloway University of London” *Should be “*Geography, Royal Holloway University of London”.

The original article has since been updated to correct these errors. Thank you for reading.
